# Chemopreventive Effects of Oplopantriol A, a Novel Compound Isolated from *Oplopanax horridus*, on Colorectal Cancer

**DOI:** 10.3390/nu6072668

**Published:** 2014-07-18

**Authors:** Zhiyu Zhang, Chunhao Yu, Chun-Feng Zhang, Xiao-Hui Wu, Xiao-Dong Wen, Samantha Anderson, Wei Du, Wei-Hua Huang, Shao-Ping Li, Chong-Zhi Wang, Chun-Su Yuan

**Affiliations:** 1 Tang Center for Herbal Medicine Research, The Pritzker School of Medicine, University of Chicago, Chicago, IL 60637, USA; E-Mails: zzhang2@bsd.uchicago.edu (Z.Z.); CYu@dacc.uchicago.edu (C.Y.); zhangchunfeng67@163.com (C.-F.Z.); longhui804@163.com (X.-H.W.); cpuwxd@gmail.com (X.-D.W.); sanderson6@uchicago.edu (S.A.); CYuan@dacc.uchicago.edu (C.-S.Y.); 2 Department of Anesthesia & Critical Care, Pritzker School of Medicine, University of Chicago, Chicago, IL 60637, USA; 3 Ben May Department for Cancer Research, Pritzker School of Medicine, University of Chicago, Chicago, IL 60637, USA; E-Mail: wei@uchicago.edu; 4 Institute of Clinical Pharmacology, Central South University, Changsha 410000, China; E-Mail: endeavor34852@aliyun.com; 5 State Key Laboratory of Quality Research in Chinese Medicine, and Institute of Chinese Medical Sciences, University of Macau, Macao, China; E-Mail: spli@umac.mo; 6 Committee on Clinical Pharmacology and Pharmacogenomics, Pritzker School of Medicine, University of Chicago, Chicago, IL 60637, USA

**Keywords:** *Oplopanax horridus*, oplopantriol A, OPT A, chemoprevention, colorectal cancer, HCT-116, SW-480, apoptosis, cell cycle, tumor necrosis factors, death receptor signaling pathway

## Abstract

*Oplopanax horridus* is a North American botanical that has received limited investigations. We previously isolated over a dozen of the constituents from *O. horridus*, and among them oplopantriol A (OPT A) is a novel compound. In this study, we firstly evaluated the *in vivo* chemoprevention activities of OPT A using the xenograft colon cancer mouse model. Our data showed that this compound significantly suppressed tumor growth with dose-related effects (*p* < 0.01). Next, we characterized the compound’s growth inhibitory effects in human colorectal cancer cell lines HCT-116 and SW-480. With OPT A treatment, these malignant cells were significantly inhibited in both a concentration- and time-dependent manner (both *p* < 0.01). The IC50 was approximately 5 µM for HCT-116 and 7 µM for SW-480 cells. OPT A significantly induced apoptosis and arrested the cell cycle at the G2/M phase. From further mechanism explorations, our data showed that OPT A significantly upregulated the expression of a cluster of genes, especially the tumor necrosis factor receptor family and caspase family, suggesting that the tumor necrosis factor-related apoptotic pathway plays a key role in OPT A induced apoptosis.

## 1. Introduction

*Oplopanax horridus* is a shrub that grows in the Pacific Northwest of North America, especially in Alaska [1]. This botanical is a member of the family Araliaceae, the same family as American ginseng. Thus, it is occasionally referred to as “Alaskan ginseng,” although they are in different genera [1,2]. Modern pharmacological studies of *O. horridus* have suggested that the botanical potentially possesses antidiabetic, antiviral, antibacterial, and anticancer activities [1,3].

**Figure 1:**
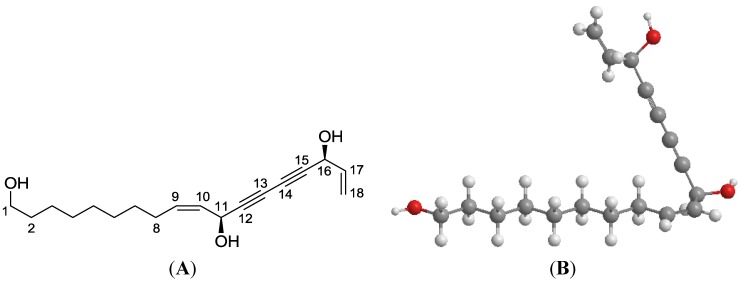
Structure models of oplopantriol A. (**A**) A two-dimensional structural formula; (**B**) a three-dimensional ball-and-stick model using spheres as atoms and sticks as bonds.

Compared to the extensive studies on ginseng (genus *Panax*), the phytochemical isolation and identification of *O. horridus* are relatively limited. The reported six polyynes isolated from *O. horridus* [4] are possibly linked to the botanical’s antimycobacterial properties. Other identified compounds include two sesquiterpenes and a polyene compound nerolidol [2,5,6]. We also reported the identification of two other polyynes, oplopantriol A and oplopantriol B, of which oplopantriol A (Figure 1) is a novel compound [4,7].

Colorectal cancer is one of the most common cancers in the West. With advanced colon cancer, the five-year survival rate is less than 10% [8,9]. Since the current available therapies for this advanced cancer have limited effectiveness, increased attention has been focused on chemoprevention. Compounds isolated from natural products contain bioactive constituents with potential health benefits, including cancer chemoprevention [10,11,12]. Using the 13 compounds which we had previously isolated and identified from *O. horridus*, we recently systemically evaluated their anticancer effects [5]. Among these compounds, oplopantriol A, the novel compound, showed significant colon cancer chemoprevention activities.

Based on the fact that many drugs used for cancer therapeutics have been developed from botanical sources, special interests have been paid to newly identified novel compounds [13]. In this study, we first evaluated whether oplopantriol A inhibited tumor growth in xenograft colon cancer mice. Next, the compound’s growth inhibition of two human colorectal cancer cells, *i.e.*, HCT-116 and SW-480 cells, were characterized. Subsequently, the underlying mechanisms of the observed colorectal cancer chemopreventive actions were explored.

## 2. Materials and Methods

### 2.1. Chemicals and Reagents

Methanol, petroleum ether (60–90 °C), ethyl acetate, *n*-butanol and DMSO were of high-performance liquid chromatography grade from Merck (Whitehouse Station, NJ, USA) or Fisher Scientific (Norcross, GA, USA). Milli Q water was supplied by a water purification system (US Filter, Palm Desert, CA, USA). Silica gel (100–200 and 200–300 mesh) was obtained from Qingdao Haiyang Chemical Co. Ltd. (Qingdao, SD, China). Reversed-phase C18 (RP-C18) silica gel (40–63 μm) was obtained from Alltech Associates, Inc. (Deerfield, IL, USA). Cell culture plasticware was obtained from Falcon Labware (Franklin Lakes, NJ, USA) and Techno Plastic Products (Trasadingen, Switzerland). Glutamine, trypsin, McCoy’s 5A and Leibovitz’s L-15 media, and phosphate buffered saline were obtained from Mediatech, Inc. (Herndon, VA, USA). Penicillin and streptomycin were obtained from Sigma-Aldrich (St. Louis, MO, USA).

### 2.2. Plant Material, Isolation and Structural Elucidation

The root bark of *Oplopanax horridus* (Sm.) Miq. from Oregon, USA was obtained from Pacific Botanicals, LLC (Grants Pass, OR, USA) and was authenticated by a botanist. The voucher specimens were deposited in the Tang Center for Herbal Medical Research at the University of Chicago. Dried root bark was ground and extracted with 80% ethanol under reflux, suspended in water, then extracted with petroleum ether (60–90 °C), ethyl acetate, and *n*-butanol. The ethyl acetate fraction was separated by silica gel, RP-C_18_ silica gel, and preparative HPLC to afford this compound. The structure of this compound was elucidated by a combination of spectroscopic analyses, including IR, ^1^H and ^13^C NMR, hydrogen–hydrogen correlation spectroscopy (H-H COSY), heteronuclear multiple quantum coherence (HMQC), heteronuclear multiple bond coherence (HMBC), mass spectroscopic data, and chemical methods. This compound was identified as oplopantriol A (OPT A, Figure 1) [7].

### 2.3. Human Cancer Cell Lines and Cell Culture

Human colorectal cancer cell lines HCT-116 and SW480 were obtained from the American Type Tissue Collection (Rockville, MD, USA) and maintained in McCoy’s 5A or L-15 medium. A HCT-116-Luc cell line that stably expresses firefly luciferase was constructed and manipulated, as described previously [14]. All medium were supplemented with 10% fetal bovine serum (FBS), penicillin (100 IU/mL), and streptomycin (100 μg/mL). The cells were subcultured twice a week and incubated at 37 °C, 95% humidity and 5% CO_2_.

### 2.4. Xenograft Tumor Xenogen Bioluminescence Imaging

Female athymic nude mice (4–6 weeks of age, Harlan, Indianapolis, IN, USA) were used. The use and care of animals were implemented with consideration of the guidelines approved by the Institutional Animal Care and Use Committee (ACUP number: 70917, approved on April 4, 2013). Before tumor cell engraftment, subconfluent HCT-116-Luc cells were harvested and re-suspended in PBS to a density of 1 × 10^7^ cells/mL. Prior to cell inoculation, cell viability was examined by a 0.4% trypan blue exclusion assay (viable cells > 90%). Approximately 1 × 10^6^ HCT-116-Luc cells in 100 μL PBS were injected subcutaneously into both flanks of each mouse for each point. Starting the same day, OPT A was administered intraperitoneally (IP) at doses of 15 or 30 mg/kg every other day, equivalent to 7.5 and 15 mg/kg/day. Control mice were injected with PBS. Animal optical imaging was carried out, as previously described [15]. Animals were exposed to the Xenogen IVIS 200 imaging system (Caliper Life Sciences, Hopkinton, MA, USA) for imaging weekly after tumor cell inoculation. d-Luciferin sodium salt (Gold Biotechnology, St. Louis, MO, USA) at 100 mg/kg in 0.1 mL sterile PBS was administered IP as a substrate before imaging. Acquired pseudo images were determined by superimposing the emitted light over the grayscale photographs of the animals. Quantitative image analysis was performed with Xenogen’s Living Image software V4.0.

### 2.5. MTS Assays

OPT A was dissolved in DMSO and stored in small aliquots at −20 °C before use. HCT-116 and SW-480 cells were seeded in 96-well plates at a density of 5000 cells/well, allowed to attach overnight and then treated with different concentrations of OPT-A (final concentration 1, 2, 4, 8, and 12 µM). Cell proliferation was measured at 24, 48, and 72 h using the Cell Titer 96 Aqueous MTS Reagent (Promega, Madison, WI, USA) according to the manufacturer’s instructions. The absorbance was read by an automated microplate reader (Epoch; Bio-Tek Instruments, Winooski, VT, USA) set to a wavelength of 490 nm [16]. Data were expressed as a ratio of treated cells *versus* control (vehicle set at 100%).

### 2.6. Apoptosis Assay

HCT-116 and SW-480 cells (5 × 10^4^) were seeded in 24-well plates. After 24 h, OPT A was applied at the indicated concentrations. After 48 h treatment, all adherent and non-adherent floating cells were harvested, and centrifuged for 5 min at 1000 rpm. Then, the cells were stained with Annexin-V (FITC) and propidium iodide (PI) (Becton Dickinson, San Diego, CA, USA) according to the manufacturer’s protocol. Double-stained cells were analyzed by a FACS Canto flow cytometer (Becton Dickinson, Mountain View, CA, USA) [17]. At least 10,000 cells were counted for analysis. All experiments were performed in triplicate, independently each time.

### 2.7. Cell Cycle Assay

For cell cycle analysis, 1 × 10^5^ HCT-116 cells were seeded in 12-well plates. On the second day, either OPT-A or DMSO (vehicle) was administrated. Treated cells continued to be cultured for 48 h. Then, all adherent cells were trypsinized, collected and fixed in 80% ethanol for 2 h at −20 °C. After being treated with 0.25% Triton X-100 for 5 min, the cells were resuspended in 50 µL of PI/RNase staining reagent (Becton Dickinson, San Diego, CA, USA), incubated in the dark for 20 min at room temperature, and finally counted with a FACS Canto flow cytometer [18]. At least 10,000 cells were read for each measurement.

### 2.8. Real-Time PCR Array of Apoptosis Screening Analysis

Cells were treated with 10 μM OPT A or vehicle for the indicated time sets (4, 12, or 24 h) and then total RNA was extracted using an RNeasy mini kit (Qiagen, Valencia, CA, USA) and quantified by Nanodrop (Thermo, Wilmington, DE, USA). The first strand of cDNA was prepared using a RT^2^ first strand kit (SAbioscience, Frederick, MD, USA). Then, the transcriptional product was analyzed using real-time PCR analysis. A human apoptosis RT^2^ Profiler PCR array plate (Cat# PAHS-012E-4, 84 key genes covered, SAbioscience, Frederick, MD, USA) was used for screening following the manufacturer’s instructions. Experiments were repeated three times. Relative gene expression levels were determined using the 2^−∆∆*ct*^ method [19].

### 2.9. Statistical Analysis

Data were expressed as mean ± SE, as indicated. Data were analyzed using Student’s *t*-test and analysis of variance (ANOVA) for repeated measures. Analyses were performed using SPSS 14.0 software (IBM Corporation, Somers, NY, USA). A *p* value set at 0.05 was used to determine the significant differences.

## 3. Results

### 3.1. Oplopantriol A Inhibited Colon Tumor Growth

**Figure 2:**
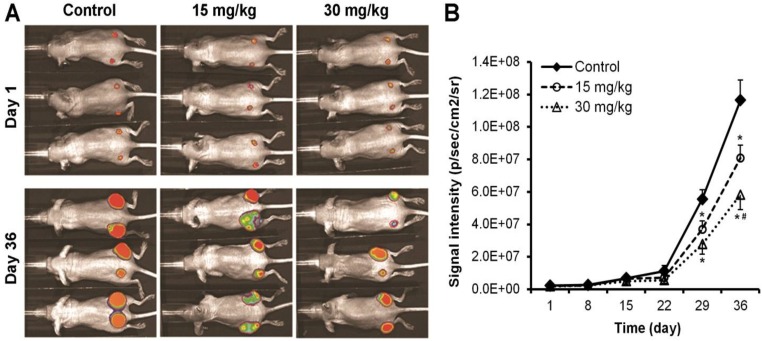
Oplopantriol A (OPT A) inhibits colon tumor growth in mice. (**A**) Human HCT-116 colorectal cancer cell xenografted tumor growth is monitored with Xenogen bioluminescence imaging assay on a weekly basis (*n* = 6). Representative Xenogen imaging at day 1 and day 36 are shown; (**B**) quantitative analysis of Xenogen bioluminescence imaging. Average tumor sizes at the indicated time points are represented with imaging signal intensities (in photons/second/cm^2^/steradian). * *p* < 0.01, compared with control; # *p* < 0.05, compared with the 15 mg/kg group.

The antitumor activity of OPT A was observed in a xenograft model using HCT-116 human colorectal cancer cells. Representative Xenogen imaging results at day 1 and 36 are shown in Figure 2A. Quantitative analysis of the imaging data is presented in Figure 2B, in which average tumor size at the indicated time points are represented by imaging signal intensities (in photons/second/cm^2^/steradian). The data showed that the OPT A treated animals exhibited significantly decreased Xenogeny imaging signals compared with those of the control group. The tumor growth inhibition was approximately 30% and 42% in 15 and 30 mg/kg dose groups, respectively. Quantitative analysis revealed that OPT A significantly inhibited xenograft tumor growth from the third week after the compound was administered (*p* < 0.01). Dose-related antitumor effects were observed in which the OPT A 30 mg/kg dose exhibited a stronger effect than the 15 mg/kg dose (*p* < 0.05). No obvious adverse events were observed in the experimental animals.

### 3.2. Oplopantriol A Inhibited HCT-116 and SW-480 Cell Proliferation

**Figure 3:**
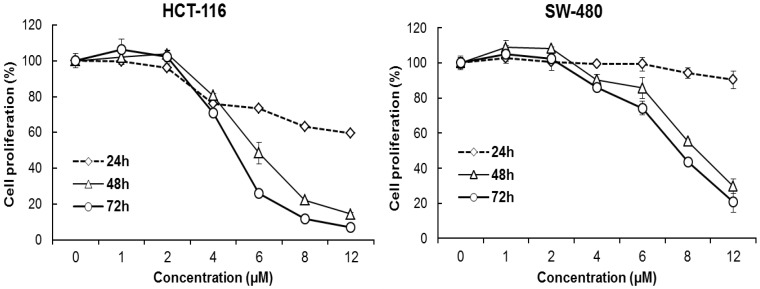
Oplopantriol A (OPT A) inhibits human HCT-116 and SW-480 colorectal cancer cell proliferation. Cell survival is determined by MTS assay and calculated as a ratio of the control. OPT A significantly inhibits HCT-116 and SW-480 cell proliferation in a time- and concentration-dependent manner (both *p* < 0.01). HCT-116 cells show more sensitivity than SW-480 cells to OPT A treatment at the indicated concentrations. Data are presented of triplicate wells and three independent experiments.

Human colorectal cancer cell lines HCT-116 and SW-480 were treated with different OPT A concentrations (1, 2, 4, 6, 8 and 12 μM) for the indicated time (24, 48 or 72 h) using the MTS assay. Figure 3 shows the concentration- and time-dependent manner decrease of cancer cell viabilities (both *p* < 0.01). The IC50 was approximately 5 µM for HCT-116 and 7 µM for SW-480 cells, and HCT-116 cells were more sensitive to OPT A treatment than SW-480 cells.

### 3.3. Oplopantriol A Induced Apoptosis

**Figure 4:**
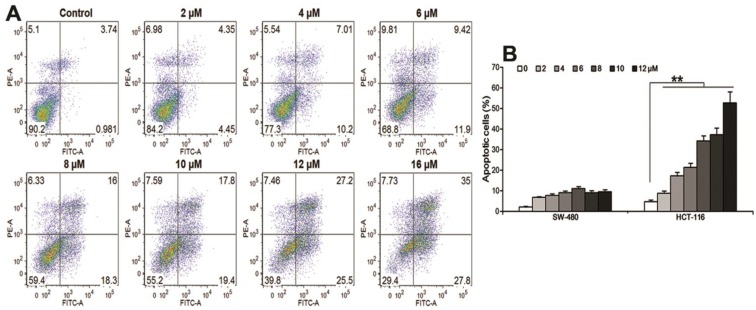
Oplopantriol A (OPT A) induces apoptosis in HCT-116 and SW-480 cells. (**A**) OPT A significantly induces apoptosis in HCT-116 cells in a concentration-related manner (*p <* 0.01); (**B**) bar graph shows that OPT A more significantly induces apoptosis in HCT-116 cells than in SW-480 cells at 48 h (** *p <* 0.01). Data are presented as a percentage of total cells counted, calculated from three observations.

Using annexin-V/PI staining, HCT-116 cells were treated with 1–16 μM of OPT A for 48 h. Apoptotic cells were captured and read by flow cytometry. The fraction of total apoptotic cells (annexin V-FITC positive) was increased in a concentration-dependent manner, *i.e.*, 4.72%–62.8% at the indicated OPT A concentrations (*p* < 0.01) in Figure 4A. Figure 4B shows that OPT A significantly induced more apoptotic cells in HCT-116 cell than in SW-480 cells at the indicated concentrations (* *p* < 0.01). This result is consistent with the cell proliferation data reported above.

### 3.4. Oplopantriol A Arrested Cell Cycle at the G2/M Phase

**Figure 5:**
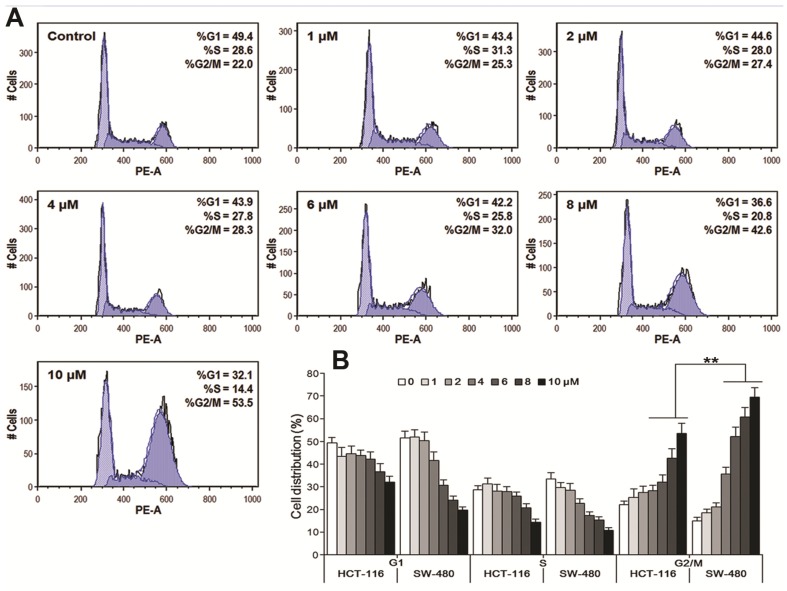
Oplopantriol A (OPT A) induces G2 cell cycle arrest in HCT-116 and SW-480 cells. Data are presented as a fraction of total cells counted. (**A**) OPT A significantly induces G2 cell cycle arrest in a concentration-related manner in HCT-116 cells (*p <* 0.01); (**B**) bar plot shows that OPT-A induces a more significant G2 cell redistribution in SW-480 cells than in HCT-116 cells, calculated from three observations (** *p <* 0.01).

HCT-116 and SW-480 cells were treated with OPT A (1–10 μM) for 48 h using PI staining to observe the compound’s effects on the cell cycle redistribution. As shown in Figure 5A, OPT A promoted G2/M cell cycle arrest in a concentration-dependent manner in HCT-116 cells (*p* < 0.01), with a G2/M cell percentage in a range of 25.3%–53.5%, compared to the control at 22.0%. Interestingly, OPT A at 6, 8, 10 μM, induced more G2/M phase captured cells in SW-480 cells, at 52.1%, 60.7% and 69.5%, than in HCT-116 cells, at 32%, 42.6% and 53.5%, respectively (Figure 5B), suggesting that the cell growth inhibition in the cell line SW-480 was induced more by the cell cycle arrest, compared to that in HCT-116.

### 3.5. Oplopantriol A Induced Apoptosis Related Gene Expression and TNF-Cell Death Pathways

To identify which signaling pathway plays the major role in controlling the phenotype exchange in programmed cell death, we examined the effect of OPT A on the expression of cell apoptosis-related genes and signaling pathways. Since a more significant apoptosis effect was observed in the HCT-116 cell line, in this study, we used the RT^2^-profiler PCR array of the cell apoptosis PCR array plate to test the effects of OPT A on the target genes in HCT-116 cells. After HCT-116 cells were treated with 10 µM OPT A for 4, 12, or 24 h, a large number of apoptosis-related genes were downregulated or upregulated compared to vehicle treated controls.

**Figure 6:**
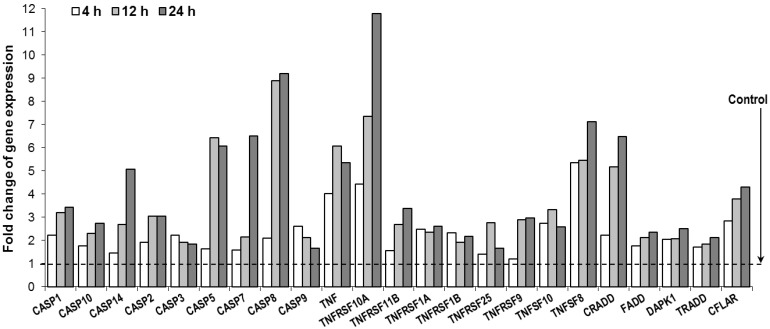
Oplopantriol A (OPT A, 10 μM) promotes changes in selected apoptosis-related genes using qPCR array. Selected transcriptional activated genes, which were involved in apoptosis induction, are shown. Genes were grouped based on gene ontology categories, which is mainly relevant to TNF-death receptor signaling pathway. Gene expression was normalized using β-actin as a control and calculated by the 2^−ΔΔ*ct*^ method.

As shown in Figure 6, OPT A significantly upregulated the expression of a cluster of genes, especially those of the tumor necrosis factor receptor family (TNF family) and caspase family, such as TNFRSF10A, TNF, TNFSF8, CRADD, FADD, TRADD, CASPSE3, 7, 8, *etc*. Notably, most of the genes showed a time-dependent effect within 24 h after OPT A exposure. This result indicated that OPT A transcriptionally activated TNF family transduction and promoted the caspases cascade downstream in the cells. Thus, the TNF-death receptor signaling pathway plays a pivotal role in the process of apoptosis trigged by OPT A treatment.

## 4. Discussion

At present, although chemotherapeutic agents have improved survival in colorectal cancer patients, the five-year survival rate is still less than 10% with the advanced disease indicating the shortcomings of currently available chemotherapeutic drugs [8,9]. Thus, there is strong rationale for exploring alternative strategies for risk reduction and treatment of this malignancy. Numerous effective drugs, including those used for cancer, have been developed from botanical sources [13,20,21]. *O. horridus* is largely not a well explored herbal medicine. In this study, we obtained and authenticated this botanical, isolated and identified a novel compound, oplopantriol A, evaluated its colon cancer chemoprevention effects using an *in vivo* antitumor mice model, and explored its related mechanisms of action.

Many murine models have been used to study human cancer. These models are developed to evaluate a response to test compounds as a first *in vivo* step. The human tumor xenograft mouse is a widely used model, in which human tumor cells can be transplanted under the skin into immunocompromised athymic nude mice that do not reject human cells [22]. In this study, using the nude mice transplanted with human HCT-116 colon cancer cells, we observed that OPT A treated animals exhibited a significant decrease in tumor size measured by Xenogeny imaging signals compared with the control group. This tumor growth inhibition began to be obvious after the third week, and over 40% tumor size reduction was shown at the end of fifth week. Dose related effects of the compound on tumor growth inhibition were also observed. For an *in vivo* test, the safety of the novel compound should also be examined. We observed no obvious adverse events in these experimental animals. When we obtain a sufficient amount of OPT A in the future, we should determine the LD50 of the compound.

Using the human colon cancer xenograft model, one can see the subcutaneous tumor growth directly, and the tumor tissue samples can be readily obtained for further investigations, such as for the tissue microarrays performed in this study. However, this orthotopic tumor athymic nude model misses the lymphocyte-mediated response to the tumor, since the nude mice lose certain T-cell responses. In addition, the nude mouse is not a gut disease-specific animal model. Therefore, it is necessary to use specific colorectal malignancy animal models, *i.e.*, AOM/DSS and Apc*^Min/+^* mouse models in our future studies [23,24,25,26]. Data obtained from different animal models can also be compared in relation to preclinical therapeutic modalities.

Our subsequent *in vitro* experiment demonstrated significant anti-proliferative effects of OPT A on two different human colorectal cancer cell lines. Each of these cell lines originated from a unique patient tumor biopsy, and has been extensively studied [27]. We used these two cell lines with genetic heterogeneity. Comparing our data of the two cell lines, HCT-116 showed a significant obvious sensitivity to OPT A more so than that shown by SW-480. In cancer chemotherapy, cancer cell apoptosis is mediated by many factors. Among them, *p*53 is a transcription factor placed at the nexus of a number of pathways that mediate apoptosis in response to a wide range of cellular stresses [28]. HCT-116 cells are *p53* wild-type, while SW-480 cells are mutant in the *p53* tumor suppressor gene. Since the effects of OPT A on HCT-116 are stronger than those on SW480, it suggests that some activity of *p53* may be linked to the compound to achieve the anti-cancer effects [29].

OPT A showed very significant antiproliferative effects in HCT-116 cells. Our data also demonstrated that OPT A significantly induced more apoptotic cells in HCT-116 cells than in SW-480 cells, consistent with the cell proliferation data. Our cell cycle assay indicated that the response to OPT A in the two cell lines was also not the same. OPT A significantly promoted G2/M cell cycle in HCT-116 cells. However, OPT A induced more G2/M phase captured cells in SW-480 cells than in HCT-116 cells. Further studies are needed to determine this discrepancy.

An apoptotic assay showed that OPT A induced HCT-116 cell apoptosis in a concentration-dependent manner. To explore the apoptotic induction mechanism of OPT A, we performed expression profiling analyses using an RT^2^-profiler PCR array containing 84 apoptotic-related genes. We observed that OPT A up-regulated the expression of several caspases in HCT-116 cells. Caspases are a family of cysteine proteases that play essential roles in cell apoptosis. This observation supported our evaluation using flow cytometry that proportions of annexin V positive HCT-116 cells were significantly increased by treatment with OPT A. Among the tested caspases, the activation of caspase 8 was most potent. Caspase 8 is a proximal effector protein of the tumor necrosis factor (TNF) receptor family apoptotic death pathway [30]. Furthermore, we observed that the expression of several TNF-related apoptosis-inducing ligand molecules were upregulated, while four genes (TNFRSF10A, TNF, TNFSF8, CRADD) were most strongly activated. These results suggest that OPT A exerts its chemopreventive effect by multiple molecular mechanisms of action on the tumor necrosis factor-related apoptotic pathway in human colorectal cancer cells.

The logical next step for signaling pathway verification would be employing western blot or immunostaining to evaluate expressions of key target regulators. Those observations may lead to the identification of markers that predict the responsiveness of colon cancer cells to OPT A treatment. This information could be used to develop OPT A derivatives as novel chemotherapeutic or chemopreventive agents for human colorectal cancer.

## 5. Conclusions

In conclusion, we isolated a novel compound OPT A from *Oplopanax horridus* root bark extract. The *in vitro* and *in vivo* effects of OPT A on human colorectal cancer were evaluated. OPT A showed significant antitumor and antiproliferative effects. In different cancer cell lines, the IC50 was between 5 and 7 µM. Cancer cell inhibition of OPT A was observed through cell cycle arrest in the G2/M phase and the induction of apoptosis. Further mechanism explorations showed that OPT A upregulated a cluster of apoptotic genes, and the tumor necrosis factor-related apoptotic pathway plays a key role in OPT A induced apoptosis.
